# Impact of Cadmium and Lead Exposure on Camel Testicular Function: Environmental Contamination and Reproductive Health

**DOI:** 10.3390/ani13142302

**Published:** 2023-07-14

**Authors:** Saif Ullah, Wael Ennab, Quanwei Wei, Changfa Wang, Abdul Quddus, Sheeraz Mustafa, Tavakolikazerooni Hadi, Dagan Mao, Fangxiong Shi

**Affiliations:** 1 College of Animal Science and Technology, Nanjing Agricultural University, Nanjing 210095, China; saifullah.vas@luawms.edu.pk (S.U.); 2016105109@njau.edu.cn (W.E.); weiquanwei@njau.edu.cn (Q.W.); haditavakoli89@yahoo.com (T.H.); maodagan@njau.edu.cn (D.M.); 2 Liaocheng Research Institute of Donkey High-Efficiency, Breeding and Ecological Feeding, College of Agronomy, Liaocheng 252000, China; wangcf967@163.com; 3 Faculty of Veterinary and Animal Sciences, Lasbela University of Agriculture Water and Marine Sciences, Uthal 90150, Pakistan; abdulquddus@luawms.edu.pk; 4 Faculty of Veterinary and Animal Sciences, Ziaddin University, Karachi 75000, Pakistan; sheeraz.mustafa@zu.edu.pk

**Keywords:** cadmium, lead, camel, testes, testosterone

## Abstract

**Simple Summary:**

The negative effects of lead (Pb) and cadmium (Cd) exposure on the testicular function of bull camels are examined in this study. We uncover the bioaccumulation of these toxic metals, which causes subcellular alterations, reduced testes, and altered hormone synthesis, through comprehensive sampling and analysis. The results highlight the significance of understanding and addressing the effects of Pb and Cd exposure on camel reproductive health.

**Abstract:**

The free grazing habits of camels from various sources may cause heavy metals to bioaccumulate in their tissues and organs, possibly resulting in higher amounts of these toxic substances in their bodies over time. The aim of this study was to assess the exposure impact of lead (Pb) and cadmium (Cd) on bull camels of the Lassi breed, aged 7 to 8 years, at a site near the industrial area and another two non-industrial sites, to analyze the presence of heavy metals. Samples from three sites were collected from thirty camels (*n* = 10/each), soil and water (*n* = 30), and five different plants (*n* = 15/each) for analysis. Testes were collected for atomic absorption spectrometry (AAS), and hematoxylin-eosin (HE) staining. Serum samples were obtained to measure testosterone levels by radioimmunoassay (RIA). Samples were obtained from plants, soil, water, blood, serum and urine for AAS. According to the results, the testes’ weight, length, width, and volume significantly decreased at the industrial site compared with the other two sites as a result of exposure to Cd and Pb. Additionally, blood testosterone concentrations were considerably lower at the industrial site, indicating a detrimental impact on testicular steroidogenesis. The histological investigation of the industrial site indicated structural disturbances, including seminiferous tubule degeneration and shedding, cellular debris in seminiferous tubules, lining epithelium depletion, and vacuolation. Elevated amounts of Cd and Pb were found at the industrial site when analyzed using water, soil, plants, testes, serum, and urine. These findings demonstrate the adverse effects of Pb and Cd exposure on camel testicular function, including decreased weight and altered steroidogenesis. These findings are essential for understanding the impact of exposure to Pb and Cd on camel reproductive function and for developing successful prevention and management plans for these exposures in this species.

## 1. Introduction

Heavy metal contamination in our environment is a serious issue for the public’s health and wellbeing. The potential harm that these hazardous compounds can inflict is made worse by the rise in heavy metal exposure, which is a result of industrialization. According to previous studies, research has connected heavy metals to a broad range of health issues, from minor to major symptoms to major long-term conditions. These issues have an impact on both personal health and the environment [1,2,3]. Industrialization is one main source that releases heavy metals into the environment, which come from natural and human sources [4]. Recently, heavy metals have attention as potentially harmful substances with a known detrimental impact on both animal and human health [5]. Numerous harmful health impacts, including reduced fertility and developmental delays, have been linked to heavy metals’ prevalence in the environment and our bodies [6,7,8]. Additionally, higher death rates have been noticed, which could be linked to the toxicity of heavy metals [9].

Industries frequently include harmful heavy metals in high concentrations, which poses a serious threat to the safe and responsible use of agricultural soil [10,11,12]. Through experiments, the harmful consequences of heavy metals have been studied, particularly in industrial settings where increased amounts of lead and cadmium have been found in the dust coming from metal-processing plants. Animals and livestock feed have also become contaminated as a result of this contamination, exposing them to these harmful compounds [13,14]. Cd and Pb can build up on the soil surfaces when industrial wastewater is utilized for irrigation over an extended period of time. This may result in a number of environmental and health problems [15]. In addition to contaminating the land, wastewater irrigation has an adverse effect on food quality and safety due to the elevated concentration of heavy metals in agricultural soils [16,17]. Due to industrial activity, harmful metals are absorbed and accumulated in both the edible and non-edible components of vegetables grown in these soils. Clinical issues in both animals and people can result from eating such metal-rich plants [15,18].

Cd is a heavy metal and a serious environmental pollutant that endangers people’s health. According to the World Health Organization (WHO), the general public is exposed to Cd through contaminants in food sources and drinking water, while occupational exposure frequently happens during industrial activities like mining or the manufacture of pigments and batteries that contain Cd [19]. This can cause contamination, which could lead to the release of Cd in the food chain [20], exposing populations to Cd through water, food and air contaminants [21]. It has been demonstrated that mammalian testes are very sensitive to Cd toxicity, according to studies [22,23,24]. Male reproductive organs might be harmed by Cd, particularly the testicles [25]. It can seriously harm adult reproductive organs like the testes, which are especially susceptible to Cd intoxication [26].

Pb is a seriously harmful environmental contaminant that can harm many different body organs. Chronic exposure to Pb can affect physiological processes and upset the balance of the oxidant-antioxidant system, which results in inflammatory responses in many tissues [27,28,29]. Pb exposure has been linked to reports of necrotic alterations in the brain, liver, and kidneys, among other organs that have been implicated in Pb toxicity [30]. Even though Pb is slowly eliminated from the body, prolonged exposure can harm numerous organs, including the testicles [31]. Studies have revealed that Pb exposure can result in impaired function of the testicles and semen quality in male animals because the testes are particularly susceptible to Pb toxicity [32,33].

Numerous studies investigating the effects of Cd and Pb on hormone levels have been conducted. Particularly, it is understood that Cd is a chemical that interferes with the endocrine system [34]. Testosterone is a crucial steroid hormone required for male sexual development and reproductive system functionality [35]. According to studies, exposure to Pb and Cd can decrease testosterone levels [36,37,38,39]. The effects of Pb and Cd poisoning on male fertility are still a topic of ongoing research.

The health of ruminants like camels is seriously threatened by the presence of Pb and Cd in the environment. Due to their grazing habits, camels are exposed to contaminated water and food sources, which could eventually cause Cd and Pb to bioaccumulate in their organs and tissues. Concern is raised about how these harmful drugs may affect their testicular histology and hormonal balance [40].

The aim of this study is to investigate the effects of Cd and Pb exposure on the testicular function of bull camels of the Lassi breed. Specifically, the study goal is to assess the impact of Cd and Pb exposure on testicular morphology, reproductive health, and steroidogenesis in bull camels grazing close to industrial and non-industrial areas. The study also analyzed the levels of Cd and Pb in environmental samples (water, soil, plants) as well as camel testes, urine, and serum. By investigating the effects of grazing in industrial and non-industrial areas, this study sheds light on the potential dangers of industrialization and its consequences on the environment and animal health.

## 2. Materials and Methods

### 2.1. Experimental Design

Soil and water samples (*n* = 30/each) were collected from three different locations in the Balochistan province of Pakistan to assess heavy metal contamination. In addition to the soil and water samples, five different types of plants (*n* = 15 for each) that camels grazed on in their surroundings were collected.

Plant A = *Dactyoctinium scindicum* (POACENE).

Plant B = *Eleusine indica* (POACENE).

Plant C = *Amaranthus viridus* (AMARANTHACEDE). Local name: Malir and Cholai.

Plant D = *Tribulus tervestrist* (ZYGOPHYLLACEAE). Local name: Gurgundo.

**Figure 1:**
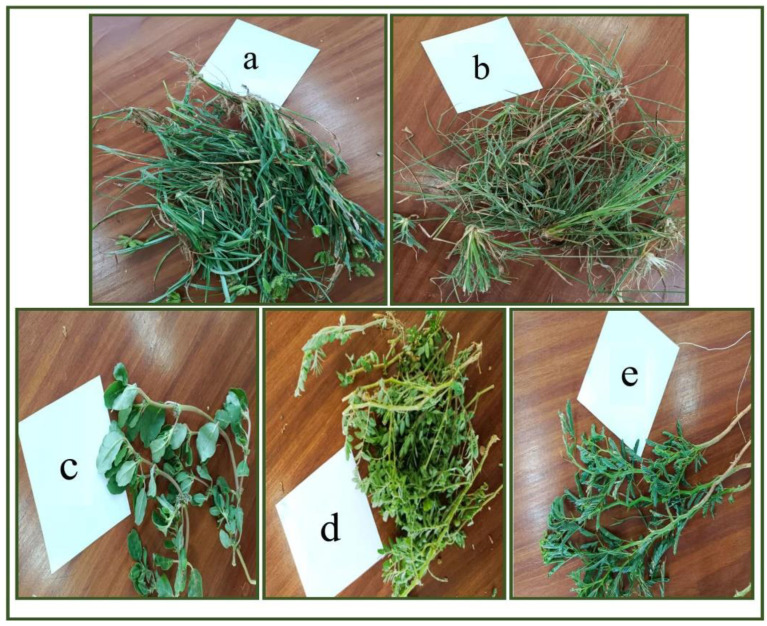
The figure illustrates five distinct types of plants, namely plants (**a**–**e**), utilized by Lassi camels for grazing at the studied sites.

Plant E = *Prospis julifora* (MIMOSACEAE). Local name: Baboor (Figure 1).

**Figure 2:**
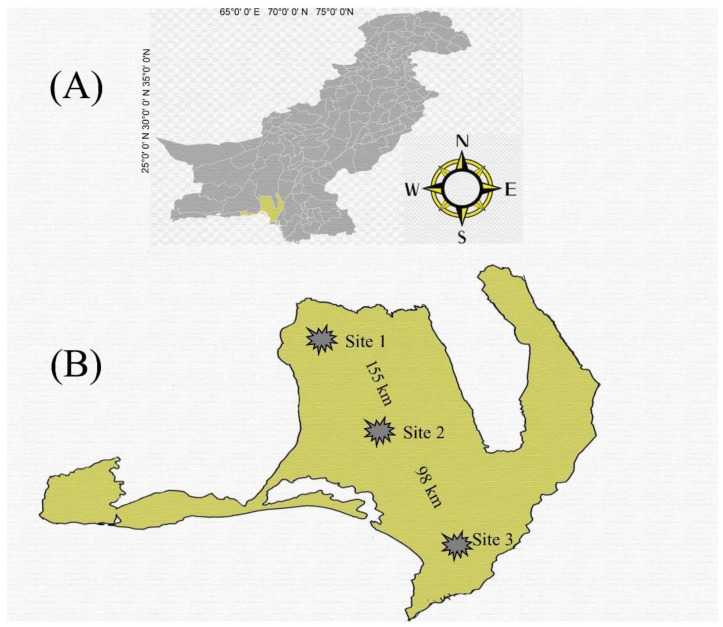
Map depicting the study area in Balochistan Province, Pakistan (**A**). The locations of the sampling sites are indicated (**B**), including the industrial site in Hub city (Site 3), Uthal city (Site 2) located 98 km away from the industrial site, and Bela city (Site 1) situated 155 km away from the industrial site.

We proposed a distance gradient from the pollution source, which is an industrial site located in Hub city. This site is referred to as Site 3, with coordinates of 25°4′3.2232″ N and 66°55′1.3368″ E. Uthal city is also considered, which is referred to as Site 2, with coordinates of 25°48′5.2″ N and 66°37′14.24″ E, and it is 98 km away from Site 3. Additionally, Bela city was also considered, which is referred to as Site 1, is non-industrial, and has coordinates of 26°13′37.56″ N and 66°18′39.96″ E. Furthermore, Site 1 served as the reference site as it was located about 155 km away from Site 3—an industrial site. (Figure 2). We chose the study area as the target area for our research based on its proximity to sources of pollution. We selected Hub city as it is situated adjacent to several factories, such as HUBCO Power Project, Bosicor Oil Refineries, Byco Petroleum Refineries, Textile Industries, Cement, Software and Telecom industries, etc., and it is considered the industrial region of the province.. The city of Bela, on the other hand, was chosen as the control area and reference site as it is agricultural land with no industrial activity and is 155 km away from the industrial site—Site 3. Additionally, Uthal city was also chosen, and it is 98 km away from Site 3.

### 2.2. Animals

Serum, urine, and testicular samples were obtained from 30 bull camels of the Lassi breed, aged 7 to 8 years, slaughtered at a local abattoir in the late autumn and early winter of the year 2021. The collected samples were categorized into three distinct groups based on their location, including Sites 1, 2 and 3, each comprising ten samples (*n* = 10). All experimental animals were healthy and fit before slaughter, and no diseased animals were used in our study. Testicular samples were obtained directly from the camels after slaughtering the animals in a slaughterhouse with full standard operating procedures (SOPs). The left testes were kept for hematoxylin-eosin (H&E) staining, while the right testes were kept for AAS.

The testes were fixed in 10% neutral buffered formalin for H&E staining analysis. In order to conduct the heavy metal analysis, testicular samples were collected, placed in clean glass containers, and kept at −20 °C. All experimental protocols were conducted in compliance with the guidelines and ethical standards approved by the Institutional Animal Care and Use Committee of Lasbela University of Agriculture, Water, and Marine Sciences LUAWMS, Pakistan, and the Nanjing Agricultural University’s Authorization Committee for Institutional Animal Care and Use (Approval Numbers: 31572403 and 31402075).

### 2.3. Testicular Size and Volume

A digital weighing scale was used to measure the weight of the individual and paired testes. Vernier calipers were used to measure the length and width [41]. Each testis was placed individually in a volume-scaled 2000 mL beaker to measure its accurate volume by the water displacement method.

### 2.4. Hormonal Assay

Blood samples were obtained following slaughtering in EDTA tubes and then centrifuged for 10 min at 4000× *g*, and for sera the supernatant was obtained. Using a commercial RIA kit (TKTT5; Diagnostic Product Corporation, Los Angeles, CA, USA), testosterone levels in the blood were measured.

### 2.5. Biochemical Analysis

Each sample was measured at 0.5 mg/mL. These samples were digested using an Anton Paar Multiwave 3000 microwave digestion system in a solution of 10 mL of HNO3 (69% Analytical Grade) and 2.5 mL of HCIO4 (30%, *v*/*v*, supra pure). The process of digestion was conducted according to the guidelines. Atomic absorption spectrometry (AAS) using a Varian SpectrAA.200 (Varian Australia, Victoria, Australia) was used to analyze the samples for cadmium and lead after they had finished being completely digested [42].

### 2.6. Histologic Assessment

Histological analysis was carried out in accordance with previous research [43,44,45,46,47]. The tissues were first dehydrated using a graded series of ethanol and xylene, followed by paraffin wax clearing and infiltration. After 24–48 h in 4% paraformaldehyde, the tissue slices were cut at a 5 µm thickness and stained with HE. Under an oil immersion microscope, 100× magnification was used to detect changes in histopathology.

### 2.7. Assessment of Seminiferous Tubules and Maturation Using Histology

The Johnsen scoring system was conducted to assess the sperm production in the seminiferous tubules. [48]. A total of thirty tubules from each cross-section were analyzed and given a score ranging from 0 to 5. The score was then recorded and described to provide a comprehensive understanding of the results obtained from the evaluation.

### 2.8. Statistical Analysis

The results were normal after they have been tested using a Shapiro–Wilk test. Graph Pad Prism 7 was utilized for the statistical analysis. The presentation of all data is as mean +/standard deviation. We used a one-way ANOVA, *t*-test, a Bonferroni’s multiple comparison post-hoc test, and Tukey’s multiple comparison to evaluate various parameters and identify individual differences. The significance level for our statistics was *p* < 0.0001.

## 3. Results

### 3.1. Quantification of Pb and Cd in Water

**Figure 3:**
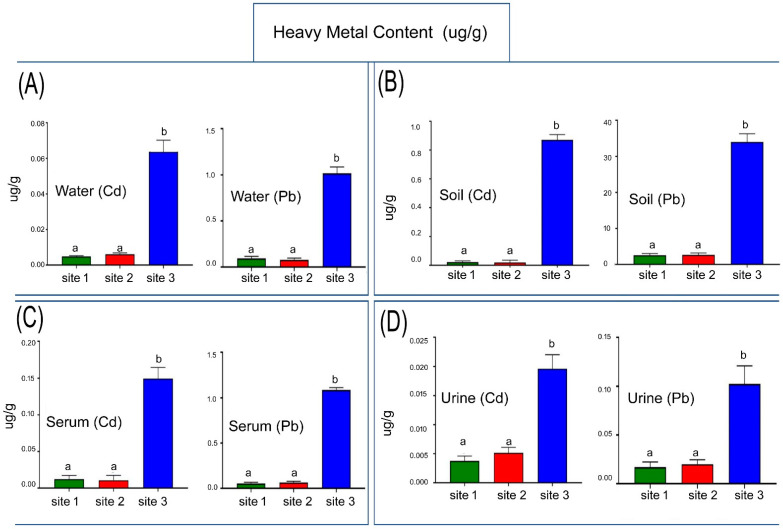
Cadmium (Cd) and Lead (Pb) concentration in the water (**A**), soil (**B**), serum (**C**) and urine (**D**) samples of studied sites. Data are mean ± SD (*n* = 10), and bars with different letters differ significantly (*p* < 0.05).

The concentration levels of Pb and Cd in water were measured in three groups: Site 1, Site 2 and Site 3. An analysis of Cd revealed an increase in water samples taken from the industrial site (Site 3) compared with the other two sites (Site 1, Site 2), (*p* < 0.0001). Pb analysis showed a significant increase in water samples obtained from the industrial site when compared with Site 1 and Site 2 (*p* < 0.0001; Figure 3A).

### 3.2. Quantification of Heavy Metals in Soil

The industrial site had a higher concentration of Cd in the soil when compared with Site 1 and Site 2 (*p* < 0.0001). Compared to Site 1 and Site 2, Pb levels in soil samples from the industrial site significantly increased (*p* < 0.0001; Figure 3B).

### 3.3. Quantification of Heavy Metals in Plants

Three sites were used in the study to measure the levels of Pb and Cd in different plants. The results revealed that plant A and plant B from Site 3 had significantly higher Cd and Pb accumulation than those from Site 1 and Site 2 (*p* < 0.0001). The Cd and Pb concentration in plant C is significantly higher at the industrial site compared with Site 1 and Site 2 (*p* < 0.0001). Compared to Site 2, Cd significantly increased at Site 1 (*p* < 0.0002). Comparing Site 3 to Sites 1 and 2, the study of Pb and Cd showed an increase in plant D and plant E (*p* < 0.0001; Table 1).

### 3.4. Quantification of Pb and Cd in Serum

The findings of this research demonstrate that Site 3 significantly increased the level of Cd in camel serum when compared to Sites 2 and 1 (*p* < 0.0001). Site 3 revealed a much higher Pb concentration when compared to Sites 1 and 2 (*p* < 0.0001; Figure 3C).

### 3.5. Quantification of Heavy Metals in Testes

**Figure 4:**
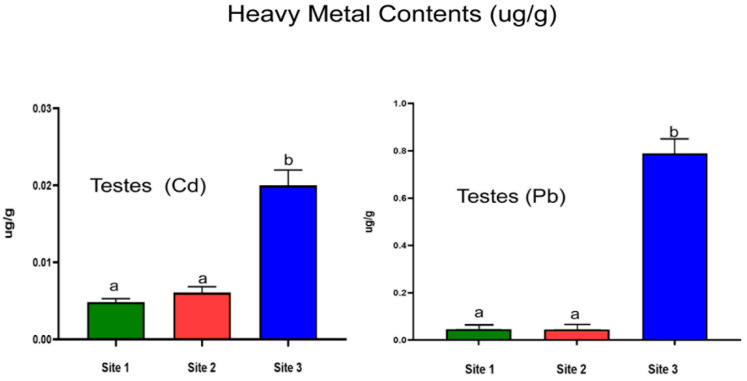
Cadmium (Cd) and Lead (Pb) concentration in testicular samples of Lassi bull camels from studied sites. Data are mean ± SD (*n* = 10), and bars with different letters differ significantly (*p* < 0.05).

Compared to Sites 1 and 2, the industrial site had significantly higher levels of Cd in testicular tissue (*p* < 0.0001). Furthermore, Pb concentrations significantly increased at the industrial site when compared with Site 1 and Site 2 (*p* < 0.0001; Figure 4).

### 3.6. Quantification of Heavy Metals in Urine

According to the study’s findings, it was found that the camel urine from the industrial site had significantly higher Cd levels than that at Site 2 and Site 1 (*p* < 0.0001). Similarly, compared to Sites 1 and 2, the industrial site’s Pb levels were significantly higher (*p* < 0.0001; Figure 3D).

### 3.7. Impact of Pb and Cd Exposure on the Testicular Dimension and Volume

The results of the study showed that exposure to Pb and Cd had a negative impact on camel testicular weight. The weight of the right, left, and paired testes at Sites 1 and 2 was significantly higher than at the industrial site, according to a statistical study. Additionally, it was shown that exposure to Pb and Cd had a detrimental effect on the testes’ size, as shown by a decrease in their length, width, and volume. When compared to the industrial site, Site 1 and Site 2 measurements of the right, left, and paired testes’ length, width, and volume were found to be significantly higher (Table 2).

### 3.8. Impact of Pb and Cd Exposure on Testicular Steroidogenesis

**Figure 5:**
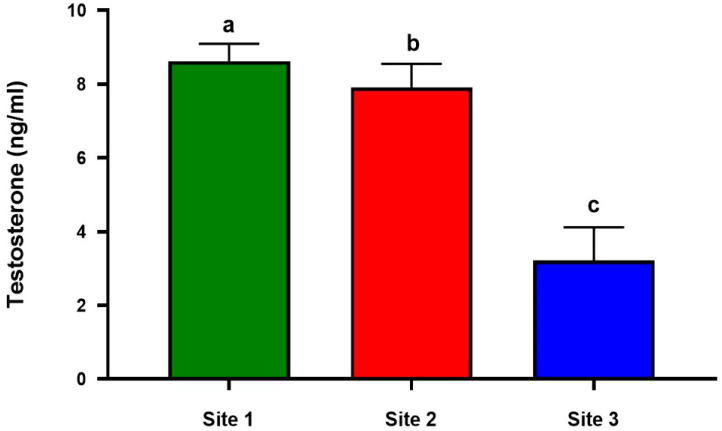
Serum concentrations of testosterone in Lassi camels at the studied sites (Site 1,2 and 3). serum concentrations of testosterone were evaluated using RIA. Values are expressed as mean ± SE (*n* = 8 for each group), and bars with different letters differ significantly (*p* < 0.05).

Serum testosterone concentrations were examined and compared between the industrial location (Site 3), Site 1, and Site 2 to determine the impact of Pb and Cd exposure. The industrial site had much lower serum testosterone levels than the other two sites, according to the statistically significant results (Figure 5).

### 3.9. The Impact of Pb and Cd Exposure on the Histological Characteristics of the Testes

**Figure 6:**
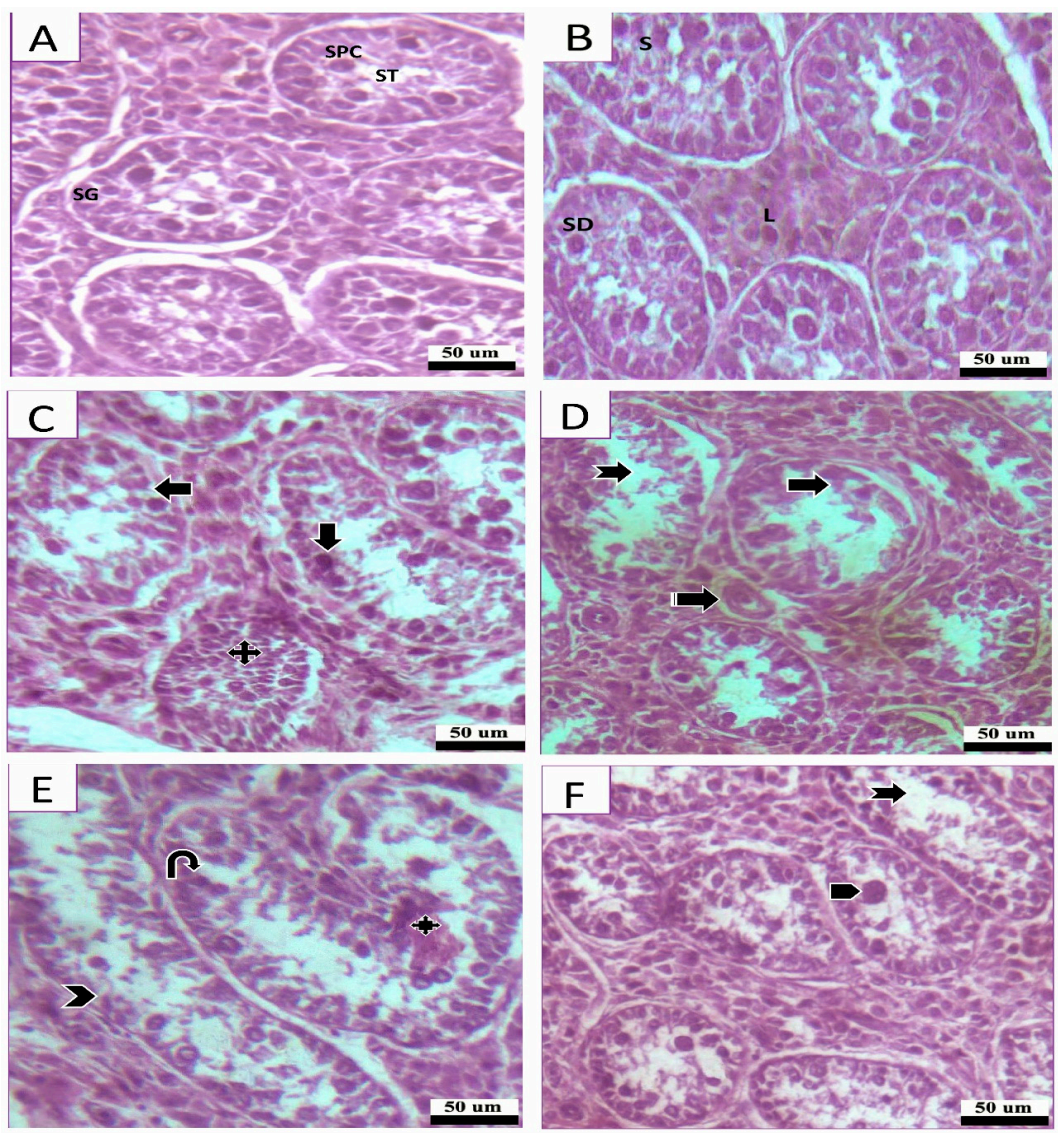
**Photomicrographs of H&E-stained testicular sections.** (**A**,**B**) A photomicrograph of a section of the testis from study sites (Site 1 and 2), displaying Sertoli cells (SC) with large vesicular nuclei and pale staining cytoplasm resting on the basement membrane and seminiferous tubules (ST) lined by many layers of spermatogenic cells organized in the form of spermatogonia (SG), spermatocytes (SPC), spermatids (SD), and sperm (S). Clusters of Leydig cells (L) can be found in the interstitial spaces. (**C**) Photomicrograph of the testis from study Site 3 (industrial site), showing the degenerating cells have darkly colored nuclei (Down arrow) and large spaces (Left arrow) between them, with a congested seminiferous tubule (Quad arrow). (**D**) Showing the reduction of the number of germ cells (Right arrow), degeneration (Notched arrow), blood vessels (Striped arrow). (**E**) Showing debris in the seminiferous tubules (Quad arrow), depletion and vacuolation (U-turn arrow), lining epithelium (Arrowhead), numerous ovoid shape (Bent arrow) Leydig cells. (**F**) Showing degeneration of seminiferous tubules (Notched arrow), formation of giant cell (Pentagon arrow).

The majority of the seminiferous tubules in Sites 1 and 2 had typical morphological characteristics, as observed through a photomicrography analysis of HE-stained histological sections (Figure 6A,B). In contrast, the industrial site showed signs of structural disruption such as tubule degeneration and shedding, cellular debris within the tubular membrane, vacuolation of cells, congested seminiferous tubules, decreased sperm production with many tubules lacking spermatozoa, disrupted cellular arrangement, fewer germ cells, and the formation of giant cells (Figure 6C–F).

### 3.10. Seminiferous Tubule Scores

In the comparison of Sites 1, 2, and 3, it was found that the industrial location had a larger count of spermatogonia, primary and secondary spermatocytes, round sperm, elongated sperm, and spermatozoa, among other stages of sperm. On the other hand, it was discovered that the average score of the seminiferous tubules at Sites 1 and 2 was much higher than that of Site 3 (Table 3 and Table 4).

## 4. Discussion

The present study was conducted to examine the effects the exposure of cadmium and lead on testicular function and structure in camels and its potential effects on animal and human health. The study discovered that soil, water, and plants, and the testicular tissue and serum of camels in the area contained significantly higher levels of Pb and Cd. These are hazardous metals that can have a variety of negative health impacts. In contrast, Pb concentrations were over 100 times the maximum permissible amount and Cd concentrations were over 14 times higher. Additionally, the soil and plants in the area contained higher concentrations of Cd and Pb than what is deemed safe by established standards. The reproductive health of both people and animals can be adversely affected by exposure to these toxic substances, especially since it alters testosterone levels and changes the structure and function of the testicles. The results of the research showed that camel testicular health was significantly impacted by exposure to Pb and Cd in contaminated and industrialized environments. In the testicular tissue and serum of camels grazing in industrial areas, the study discovered a significant rise in contents of Pb and Cd. The increased concentrations of Pb and Cd in the testicular tissues suggested that exposure to these heavy metals could affect testicular function and even lower testosterone levels in camels. The study also found that exposure to Pb and Cd was linked to a reduction in testicular weight, size, and volume. Reduced fertility and probable population losses in camels could result from these changes in testicular structure and size that affect the ability of the testicles to create and preserve healthy sperm. When camels are exposed to certain heavy metals, their fertility may be lowered and their testosterone levels may drop, which may contribute to camel population decreases. Furthermore, even at low amounts, prolonged exposure to Pb and Cd can alter the histopathology of camel tissues.

The concentration of heavy metals in water revealed that the industrial site had much higher levels of Pb and Cd, which are toxic and harmful to both animal and human health. For the sake of ensuring public health and safety, the safe levels of Cd and Pb in water are crucial factors. The National Standards (China MH&NS), the World Health Organization (WHO), and the Chinese Ministry of Health have suggested safe limits of 0.005 mg L^−1^ for Cd and 0.01 mg L^−1^ for Pb in water [49,50]. Similarly to this, the US Environmental Protection Agency (USEPA) recommended safe water limits of 0.005 mg L^−1^ for Cd and 0.05 mg L^−1^ for Pb [51]. However, according to the results of our study, the water at the industrial site is more contaminated than is considered safe with Pb and Cd. Cd levels in the water were specifically found to be 0.07 mg L^−1^, which is more than 14 times the permissible limit. Similar to Pb, the concentration was found to be 1.25 mg L^−1^, more than 100 times the permissible limit. Considering that exposure to Cd and Pb in water has been connected to several harmful health effects, including damage to kidneys, liver, and the nervous system in addition to developmental and reproductive problems, these findings suggest that the water is significantly contaminated and could pose a significant risk to human and animal health [52,53].

Heavy metals such as Pb and Cd are frequently present in high concentrations in the soil in industrial locations. Cd and Pb are typically found in soils in low amounts and are not necessary for plant growth. These heavy metals can, however, be discharged in significant amounts into the environment as a result of human activities like smelting, mining, and industrial processes, contaminating adjacent soils and water sources. As a result, there may be elevated Pb and Cd levels in the soil around industrial regions, which could be toxic to both environmental and human health. [54]. Depending on the concentrations, exposure to Pb and Cd in soil and plants can result in major health risks, including damaged liver, kidney, and reproductive systems [52,53]. Safe limits have been set for Pb and Cd concentrations in soil and plants by several organizations, including the World Health Organization (WHO) and the Chinese government. According to WHO, the permissible levels of Cd and Pb in soil are 0.003 ug/g and 0.1 ug/g, respectively. The Chinese standard for soil Cd and Pb concentrations, meanwhile, ranges between 0.3 and 0.6 ug/g and 80 ug/g, respectively. The WHO establishes a similar level of 0.02 ug/g for Cd and 0.3 ug/g for Pb for plants, while the US Environmental Protection Agency (EPA) recommends a safe limit of 0.02 ug/g for both [55,56]. In industrial sites, our study observed higher contamination levels of Cd and Pb in the soil and plants. Particularly, 0.88 ug/g of Cd and 32 ug/g of Pb were discovered to be present in the soil. Cd accumulation in plants varied from 0.89 to 0.127 ug/g, whereas Pb accumulation ranged from 0.86 to 2.28 ug/g. According to these findings, the soil and plants in the industrial site are more likely to contain more Cd and Pb than is safe according to established standards, which could be dangerous for the health of both animals and the environment.

Heavy metal concentrations in the testicular tissue and serum were measured, and it was discovered that the industrial site had much higher levels of Cd and Pb. Pb and Cd exposure can be harmful to fertility and reproductive health. Numerous studies, including one that discovered elevated levels of Cd and Pb in cow milk that was collected from areas near industrial areas, support this [57]. Another study discovered that the accumulation of Cd and Pb in camels’ liver and kidney was linked to functional and histological changes, altered kidney and liver function, apoptosis, oxidative stress and tissue damage [58]. Even at low levels, prolonged exposure to Pb and Cd can alter the histopathology of rodent tissues [59], and changes to spermatogenesis, morphological changes, decreased testosterone levels, and impairments in the function of reproductive organs [60]. Reduced sperm quality brought on by this exposure can also lower fertility. In particular, cadmium exposure can cause oxidative stress and result in the death of sperm cells, which can reduce testosterone production and affect fertility [61]. It has been demonstrated that cadmium harms testicles in animals and humans [62]. According to studies, exposure to cadmium can affect the testicular structure, resulting in histopathological changes and lower testosterone levels in mice [63,64,65,66]. Recent research highlighting the harmful effects of Cd exposure on male reproductive health confirmed these findings [67].

Scientific research has proven the harmful impacts of Cd exposure on the health of male reproduction, including the finding that Kermani sheep living close to industrial areas experienced negative impacts from long-term exposure related to histology, size of testes, and sperm parameters [68]. These results demonstrate the need for limiting Cd exposure to protect male fertility and reproductive health. Additionally, studies have demonstrated that Pb and Cd, either separately or together, have a detrimental effect on the process of testicular steroidogenesis [63,69,70]. Male reproductive health has been observed to decline as a result of exposure to Cd in numerous studies. According to research, animals exposed to even low amounts of Cd can experience testicular disruption and lower testosterone levels in rodents [71,72]. This finding was supported by a study conducted by Zhu, Q. et al. (2020), which highlights the detrimental impact of Cd exposure on the reproduction of males [67]. Our results in camels are consistent with earlier studies that indicate even low levels of Cd exposure can cause histological changes in the testes, such as lower testosterone levels and damage to the seminiferous epithelium. These modifications show the interrelated connection between Cd exposure and male infertility and are brought on by the impairment of testicular essential metal homeostasis [64,73,74]. These results highlight the significance of minimizing exposure to heavy metals, particularly Cd, to protect the health of male reproduction. Scientific research has shown that lead and cadmium, either separately or together, have a detrimental effect on the process of testicular steroidogenesis.

Pb is a toxic heavy metal that can have a detrimental effect on individuals as well as the environment. Studies have indicated that lead exposure can change the structure and function of these tissues, especially in camels that graze close to industrial sites, and can raise the levels of lead in the meat, liver, kidney, milk and blood [58]. Research has shown that lead can have a detrimental effect on male reproductive health by reducing fertility due to decreased sperm quality and impaired spermatogenesis in the testes, disrupting the process of testicular steroidogenesis and causing a variety of negative health effects [75,76,77]. Lead exposure can affect reproduction even at low doses, harming sperm and the testes cellular structure [78,79], reducing testosterone levels, altering sperm morphology, and causing oxidative stress in rats [80,81]. An increase in Pb content near industrial areas has been linked to alterations in the structure of the testes and decreased fertility in rams [68]. According to our research, exposure to cadmium and lead accumulation can further alter the structure of the testicles and lower serum testosterone levels.

Androgens are a class of steroid hormones necessary for the proper function of some reproductive tissues, including the testes [82]. With the testes producing over 95% of the hormone, testosterone is a dominating androgen in males [83]. It has been shown that exposure to heavy metals like lead and cadmium has a negative impact on testosterone levels in humans and animals. According to numerous studies, exposure to these toxic substances can result in a reduction in testosterone secretion and synthesis as well as a reduction in testicular function [14,84,85]. The current study’s goal was to evaluate the effects of heavy metal exposure in an industrial site on testosterone level in camels. The impact of heavy metal exposure in an industrial site on the testosterone level in camels was assessed by our findings. The findings revealed a significant reduction in testosterone in bull camels exposed to heavy metals. Previous research, such as that conducted by Heidari, A.H., et al. (2021), has also demonstrated that exposure to heavy metals can change testosterone production in other animals, such as rams, indicating that the harmful effects of heavy metals may interfere with the delivery and metabolism of cholesterol—a vital component in testosterone synthesis [68].

Research has shown that exposure to these metals in rats, mice, and numerous animal species resulted in lower testicular weight as well as decreased testosterone levels and spermatogenesis. Exposure to Cd and Pb has also been demonstrated to impair testicular size and volume and affect male reproductive health [86,87]. According to our research, camels’ testicles have been similarly affected in terms of size and volume by heavy metal exposure. The results of this study demonstrate the harmful impact of heavy metal exposure on camels’ testicular structure and function. These findings highlight the need to limit heavy metal exposure to protect the health of both animal and human populations. The study of heavy metals in urine showed that Cd and Pb measurements were much higher in the industrial group. Exposure is still a major problem, even though this indicates that the body is removing the heavy metals through urine. More research is needed to completely comprehend the processes through which exposure to heavy metals impair testicular function and to develop effective mitigation strategies.

## 5. Conclusions

In conclusion, our research shows that camels grazing freely in industrial regions may cause heavy metal accumulation in their organs and tissues, which could have a negative impact on their ability to reproduce. The weight, size, and volume of the testes, as well as the production of testosterone and sperm, have all been demonstrated to be dramatically reduced by exposure to cadmium and lead. Furthermore, seminiferous tubule degeneration and shedding, as well as the presence of cellular debris and vacuolation, were observed by histological investigation. The soil and water in industrial regions, as well as the plants and camel serum samples, were found to have significantly higher levels of cadmium and lead. These findings demonstrate the need for efficient ways to avoid and control heavy metal exposure in camels, as well as the possible health hazards linked with environmental pollution in industrial regions. Overall, this study’s findings highlight the significance of monitoring camel exposure to heavy metals, especially in those who graze close to industrial regions, and putting protective measures in place to reduce such exposure. Such efforts are essential for preserving this important species’ reproductive health and general wellbeing.

## Figures and Tables

**Table 1:** Cadmium (Cd) and Lead (Pb) concentration in five different plants from studied sites. Data are mean ± SD (*n* = 10), and different letters differ significantly (*p* < 0.05).

Cd (ug/g)				Pb (ug/g)		
	Site 1	Site 2	Site 3	Site 1	Site 2	Site 3
**Plant A**	0.010 ^a^	0.014 ^a^	0.137 ^b^	0.714 ^a^	0.698 ^a^	2.283 ^b^
**Plant B**	0.008 ^a^	0.006 ^a^	0.089 ^b^	0.061 ^a^	0.083 ^a^	1.209 ^b^
**Plant C**	0.035 ^a^	0.007 ^b^	0.129 ^c^	0.235 ^a^	0.302 ^a^	2.091 ^b^
**Plant D**	0.010 ^a^	0.013 ^a^	0.110 ^b^	0.098 ^a^	0.116 ^a^	0.869 ^b^
**Plant E**	0.008 ^a^	0.010 ^a^	0.097 ^b^	0.074 ^a^	0.088 ^a^	0.938 ^b^

**Table 2:** Anatomical results of Lassi camel testes from studied sites, (a–h) Means (*n* = 10) with different letters within the column are significantly different (*p* > 0.05).

Testes/Studies	Factor	Right Testes	Left Testes	Paired
**Weight (g)**	**Site 1**	94.7 ± 13.5 ^a^	92.3 ± 12.8 ^a^	187 ± 26.4 ^a^
	**Site 2**	93.9 ± 14.6 ^a^	90.9 ± 14.3 ^a^	185 ± 27.8 ^a^
	**Site 3**	81.1 ± 12.5 ^b^	79.4 ± 11.2 ^b^	160.5 ± 22.5 ^b^
**Length (mm)**	**Site 1**	87.3 ± 12.4 ^c^	85.6 ± 11.4 ^c^	
	**Site 2**	88.2 ± 12.1 ^c^	84.8 ± 12.5 ^c^	
	**Site 3**	74.8 ± 11.3 ^d^	74.2 ± 10.5 ^d^	
**Width (mm)**	**Site 1**	57.3 ± 11.8 ^e^	55.5 ± 14.3 ^e^	
	**Site 2**	55.4 ± 12.5 ^e^	56.6 ± 12.8 ^e^	
	**Site 3**	45.4 ± 9.9 ^f^	44.3 ± 12.5 ^f^	
**Volume (cm^3^)**	**Site 1**	141.3 ± 14.3 ^g^	139.3 ± 13.9 ^g^	
	**Site 2**	140.7 ± 14.9 ^g^	141.0 ± 12.5 ^g^	
	**Site 3**	127.2 ± 13.8 ^h^	125.4 ± 12.2 ^h^	

**Table 3:** Criteria of scores for the evaluation of Lassi camel spermatogenesis from studied sites.

Groups	Number of Sections	Score					
		5	4	3	2	1	0
**Site 1**	30	(80%) 25	(36%) 11	(50%) 15	(20%) 6	(27%) 8	(13%) 4
**Site 2**	30	(77%) 23	(34%) 10	(53%) 16	(26%) 8	(24%) 7	(13%) 4
**Site 3**	30	(40%) 12	(16%) 5	(10%) 3	(46%) 14	(33%) 10	(13%) 4

**Table 4:** Frequency and percentage of seminiferous tubule score 5, 4, 3, 2, 1, and 0 in multiple cross-sections in studied sites.

Score	Description
5	Complete spermatogenesis with mature sperm cells
4	Some sperm cells, with a disorganized epithelium
3	Presence of few sperm (<5 to 10)
2	Absence of sperm cells, presence of spermatids
1	Absence of sperm cells, presence of a few spermatids
0	Absence of sperm cells or spermatids, presence of spermatocytes

## Data Availability

Not applicable.
